# The intensity of informal caregiving and its implications for older caregivers: a national survey in Sweden

**DOI:** 10.1177/14034948251335113

**Published:** 2025-05-01

**Authors:** Mariam Kirvalidze, Elizabeth Hanson, Lennart Magnusson, Lena Dahlberg, Anders Wimo, Lucas Morin, Amaia Calderón-Larrañaga

**Affiliations:** 1 Aging Research Center, Department of Neurobiology, Care Sciences and Society, Karolinska Institutet and Stockholm University, Solna, Sweden; 2 Swedish Family Care Competence Centre (Nka), Kalmar, Sweden; 3 Department of Health and Caring Sciences, Linnaeus University, Kalmar, Sweden; 4 School of Health and Welfare, Dalarna University, Falun, Sweden; 5 Division of Neurogeriatrics, Center for Alzheimer Research, Department of Neurobiology, Care Sciences and Society, Karolinska Institutet, Solna, Sweden; 6 Department of Medical Epidemiology and Biostatistics, Karolinska Institutet, Solna, Sweden; 7 High-Dimensional Biostatistics for Pharmacoepidemiology and Genomics, Center for Epidemiology and Population Health, Inserm, Paris, France; 8 Stockholm Gerontology Research Center, Stockholm, Sweden

**Keywords:** Informal care, aging, caregivers

## Abstract

**Background:**

Informal caregiving is a crucial—albeit often invisible—part of the support system that enables older people with chronic diseases, disability, or age-related conditions to live in the community. However, providing informal care can affect caregivers’ lives.

**Aims:**

To explore 1) the level of care intensity among older caregivers, 2) the relationship between the intensity of caregiving and the negative experiences reported by caregivers, and 3) the variations in unmet support needs depending on the intensity of caregiving.

**Methods:**

Between May and September 2023, we conducted a national representative survey to map informal caregivers in Sweden. A total of 25,776 older adults aged ⩾65 years were sampled. Marginal probabilities were calculated to obtain results adjusted for age, sex, and level of education.

**Results:**

A total of 15,129 people aged ⩾65 years responded to the survey (58.7%), of which 2157 were informal caregivers (14.3%). During a typical week, 68.6% of caregivers provided 1–10 h of informal care, 14.6% provided 11–29 h, and 16.8% provided at least 30 h of care. Women (63.1%) and caregivers aged ⩾75 years (64.1%) were overrepresented in the group providing high-intensity informal care. A higher intensity of care was related to reporting more negative experiences and worse health, as well as to experiencing more unmet support needs.

**Conclusions:**

While most older informal caregivers reported low-intensity engagement and overall good satisfaction with their situation, a non-negligible fraction provides high-intensity help and has unmet needs that should be addressed by targeted interventions rather than one-size-fits-all policies.

## Background

Informal caregiving, often an invisible part of health and social care, plays a crucial role in supporting individuals with chronic illnesses, disabilities, or age-related conditions within their homes and communities. Estimates suggest that between 10% and 25% of Europe’s population regularly engage in informal caregiving [1]. The extent of caregiving by older people is less known, despite expectations that this group will increasingly assume a significant share of caregiving responsibilities in the future [2,3].

The intensity of caregiving (the frequency and duration of care-related tasks) varies greatly between individuals, from providing social support 1 h a day to providing round-the-clock care [4]. A higher intensity of informal care increases the burden on caregivers, and thus may lead to a greater risk of negative health and well-being outcomes among those providing informal care. Indeed, studies have shown that high-intensity caregiving can negatively affect the mental and physical health of caregivers [5,6], as well as decrease their quality of life [7]. In addition, older informal caregivers might be at a higher risk of negative outcomes owing to their own age-related problems [8,9]. However, previous studies have also shown that some caregivers may experience a sense of purpose and meaningful engagement from care receivers, even in high-intensity care situations such as dementia care [10–12].

In Sweden, with a welfare state known for its generous social policies, informal caregivers still play a vital role in supplementing formal healthcare and social care services [13]. However, the specific needs and challenges faced by older caregivers have been underexplored. While previous research in Sweden has identified negative health outcomes due to feeling burdened by informal care [12,14,15], there is a lack of research based on representative samples of older caregivers. Instead, studies often either grouped younger and older caregivers together, imposed restrictive upper age limits, or focused on carers of a specific group such as persons with dementia or cancer, thereby overlooking the experience of the growing number of older caregivers who care for a spouse, sibling, centenarian parent and/or adult offspring with health and/or care needs, while managing their own health issues.

In addition, we do not know whether caregivers receive adequate and suitable support from public authorities. According to the 2009 amendments to the Social Services Act, informal caregivers in Sweden are entitled to receive support from the municipalities in which they live [16]. The latest available audit report on caregiver support services identified both a lack of flexibility and variable quality of delivered services across municipalities [17]. There are major policy and societal changes related to informal care in Sweden. Notably, the country launched its first National Strategy for Caregivers in 2022, which outlines the definitions of informal care, urges researchers to better understand the needs of caregivers, and acknowledges that informal care can become overly extensive and negatively impact caregivers [18]. This strategy also acknowledges the need for more up-to-date statistics on informal care. Thus, a recent survey conducted by Karolinska Institutet and the Swedish Family Care Competence Centre, in collaboration with Statistics Sweden, provides an opportunity to map out the current landscape of informal care among adults aged 55 years and older across Sweden.

Our study presents the first results from this survey, focusing on a subsample of caregivers 65 years and older, a population group that is often only seen as receivers of informal care and formal care services. In this paper, we aim to examine the level of intensity of informal caregiving and its relationship with negative experiences, self-rated health, and unmet support needs reported by older informal caregivers.

## Methods

### Participants

This study focuses on a subgroup (older informal caregivers aged 65 years and older) of a nationally representative cross-sectional survey of people aged 55 years or older in Sweden. The sampling frame was created using data from the Total Population Register, which covers the entire Swedish population, and stratified according to age, education, country of birth, and region of residence. Based on an expected response rate of ~60% and a hypothesized proportion of 15% of caregivers among the respondents, informed by previous surveys, we calculated that at least 30,000 people would have to be included to obtain the desired level of precision (±1 percentage point around the prevalence estimate). To allow for appropriately powered subgroup analyses, people aged 65 years and older and those living in smaller or less densely populated regions were oversampled. Sampling weights were then calculated to produce nationally representative estimates of the proportion of caregivers in the general population of the relevant age group. Differences in sex, age, and other sociodemographic variables between responders and non-responders of the entire sample are presented in Supplementary Table 1. The flowchart depicting sample selection is presented in Supplementary Figure 1.

### Main exposure and outcomes of interest

The exposure and main stratification axis of this study was the intensity of the care provided. To derive this variable, we used the question “On average, how many hours per week do you provide care (to one or more persons, altogether)?” The low-intensity group was determined as persons responding, “less than one hour per week” or “1–10 hours per week.” The moderate-intensity group was designated as responders to “11–29 hours per week.” Finally, the high-intensity group comprised responses “30–59 hours per week” or “60 hours or more per week.” These categories were assigned based on the previous literature and the distribution of our data. There were also more detailed questions about the number of hours per week under the framework of the Resource Utilization in Dementia [19] and about the kind of support the caregivers provided. The outcomes included perceived disturbances to life, need for support, and self-rated health. Both the original Swedish and translated English versions of the questionnaire are provided in Supplementary File 1.

### Data collection

Data on the sociodemographic characteristics of the sample (age, sex, education, income, region of residence, characteristics of the residential area, birthplace, and civil status) were obtained from the Total Population Register. The survey was conducted between May and September 2023 and was administered by Statistics Sweden. Participants were first sent a postal invitation with a leaflet containing detailed information about the survey, a link and information on how to complete the survey online, and a printed version of the questionnaire. Paper-based questionnaires were mailed twice, followed by two reminders in between, each containing online log-in information. The questionnaire, consisting of 25 structured questions, was designed and piloted by the research group and was loosely based on two previous surveys [20,21] and previous research. To boost the response rate, our research group devised a dissemination strategy in partnership with the Karolinska Institutet Press Office and the Swedish Family Care Competence Centre, using relevant outlets such as newspapers, blogs, and radio.

### Identification of informal caregivers

The questionnaire included an introductory text on what defines informal care in this survey (e.g., excluding care for healthy grandchildren, see Supplementary File 1), followed by two screening questions for identifying caregivers. First, respondents answering positively to “Do you regularly (i.e., not just temporarily for a short period) provide care or support to one or more people?” were selected. Second, “How often do you provide care or support (to one or more people)?” was asked with possible options “Every day,” “At least once a week,” “At least once a month,” and “Less than once a month.” Those responding “Less than once a month” were excluded. This was done to filter out very occasional caregivers, who probably do not share the experiences of regular caregiving.

### Statistical analysis

We report descriptive findings for all sections of the survey, stratifying the analyses by the intensity of informal care provision. In addition to crude (unadjusted) outcome measures, we computed marginal probabilities from logistic regression models in order to balance basic demographic characteristics (age, sex, and level of education) across levels of intensity of caregiving, related to all outcomes (experiences, support needs, and self-rated health). Since variables used in the analyses had less than 15% missing responses, complete case analyses were performed. We conducted a sensitivity analysis to compare two questions depicting the intensity of caregiving. Namely, in addition to the aforementioned question on average hours per week, the questionnaire also included several questions on time spent on specific care tasks (such as personal care, supervision, social support). We compared the time distribution between the two questions. Statistical analyses were performed using StataSE 15 (StataCorp, College Station, TX).

### Reporting and ethical approval

The reporting of the survey methodology includes information on the relevant items of the Preferred Reporting Items for Complex Sample Survey Analysis (Supplementary Table 2) [22]. Ethical approval was granted prior to data collection from the Swedish Ethical Review Authority (2022-06383-01).

## Results

The survey was sent out to 25,776 people aged 65 year or older, yielding a total of 15,129 participants (response rate: 58.7%). From these, 2157 respondents (14.3%) reported being involved in informal caregiving, of which 2102 provided information on the intensity of provided care. The numbers and proportion of caregivers across the entire survey sample are presented in Supplementary Table 3.

In our analytical sample, the intensity of care was highly variable: during a typical week, 68.6% of caregivers provided 1–10 h of informal care, 14.6% provided 11–29 h, and 16.8% provided at least 30 h of care. The characteristics of caregivers as a group, and caregivers in each of these three intensity-based groups are reported in Table I. Briefly, women (63.1%) and caregivers aged ⩾75 years (64.1%) were overrepresented in the high-intensity category. Caregivers providing more than 30 h of care per week had a mean age of 77.5 years, compared with a mean age of 74.3 years in the group providing 1–10 h of care. Hence, 227 out of 1025 (22.1%) caregivers aged 75+ provided at least 30 h of informal care, compared with just 11.8% of those aged 65–74 years. Spousal caregiving was the predominant type of high-intensity caregiving, with 83.6% of high-intensity caregivers providing care to a spouse. The most common reasons for providing informal care reported by high-intensity caregivers were dementia (36.2%) and physical limitations (39.3%).

**Table I.** Characteristics of informal caregivers and care receivers, provided for the entire analytical sample (all informal caregivers over the age of 65 years, *N* = 2157) and according to the number of hours of informal care provided per typical week.

Number (%) of caregivers	All caregivers	Intensity of caregiving (hours/week)*		
		1 to 10 h	11 to 29 h	⩾30 h
	2157 (14.2)	1441 (68.6)	307 (14.6)	354 (16.8)
Sex, *n* (%)				
Male	912 (44.4)	630 (44.2)	130 (43.2)	130 (36.9)
Female	1245 (55.6)	795 (55.8)	171 (56.8)	222 (63.1)
Age, years (%)				
Mean (SD)	75.3 (6.9)	74.3 (6.8)	76.4 (6.9)	77.5 (6.8)
65–74	1086 (54)	818 (56.8)	132 (43)	127 (35.9)
75–85	830 (33.9)	497 (34.5)	132 (43)	172 (48.6)
>85	241 (12.1)	126 (8.7)	43 (14)	55 (15.5)
Birthplace, *n* (%)				
Born outside Sweden	239 (18.3)	130 (9)	40 (13)	57 (16.1)
Born in Sweden	1918 (81.7)	1311 (91)	267 (87)	297 (83.9)
Highest educational attainment, *n* (%)				
Lower than high school	374 (20.1)	232 (16.1)	52 (17)	72 (20.5)
High school	937 (47.4)	631 (43.9)	128 (41.8)	155 (44.2)
Higher education	839 (32.5)	575 (40)	126 (41.2)	124 (35.3)
Retirement status, *n* (%)				
Fully retired	1811 (81.5)	1173 (83.4)	265 (87.2)	326 (93.1)
Working	98 (8.9)	85 (6)	7 (2.3)	6 (1.7)
Retired, working part-time	189 (8)	143 (10.2)	29 (9.5)	16 (4.6)
On sick leave	3 (0.4)	2 (0.1)	0 (0)	1 (0.3)
Voluntarily not working	12 (1.2)	3 (0.2)	3 (1)	1 (0.3)
Civil status, *n* (%)				
Married or partnered	1428 (61.5)	911 (63.2)	211 (68.7)	263 (74.3)
Not married	237 (12.6)	173 (12)	35 (11.4)	29 (8.2)
Divorced	332 (18.2)	246 (17.1)	43 (14)	37 (10.5)
Widow(er)	160 (7.7)	111 (7.7)	18 (5.9)	25 (7.1)
Quartiles of disposable income per household, *n* (%)				
Lowest (up to 250,000 SEK)	857 (40.6)	518 (35.9)	127 (41.4)	177 (50)
2nd (251,000–415,000 SEK)	818 (37.7)	559 (38.9)	120 (39.1)	121 (34.2)
3rd (416,000–669,000 SEK)	349 (16.1)	267 (18.5)	43 (14)	38 (10.8)
Highest (670,000+ SEK)	133 (5.7)	97 (6.7)	17 (5.5)	18 (5)
Primary care receiver, *n* (%)				
Spouse	1028 (48)	473 (32.9)	213 (69.4)	309 (87.3)
Child	195 (9.1)	133 (9.2)	39 (12.7)	21 (5.9)
Parent	475 (22.2)	428 (29.7)	30 (9.8)	16 (4.5)
Sibling	107 (5)	96 (6.7)	10 (3.3)	1 (0.3)
Relative	157 (7.3)	146 (10.1)	7 (2.3)	4 (1.1)
Friend	177 (8.3)	163 (11.3)	8 (2.6)	3 (0.8)
Care receiver’s sex, *n* (%)				
Male	927 (44)	541 (37.8)	151 (49.7)	206 (58.7)
Female	1196 (56)	887 (61.9)	153 (50.3)	145 (41.3)
Care receiver’s age, years				
Mean (SD)	78.6 (16.8)	79.9 (16.9)	74.6 (16.4)	77.5 (13.6)
Care receiver’s living place**, *n* (%)				
Own housing	1723 (81.5)	1078 (75.8)	277 (90.8)	333 (96)
Housing for older persons	81 (3.8)	72 (5.1)	2 (0.7)	5 (1.4)
Serviced housing for older persons	47 (2.2)	40 (2.8)	6 (2)	0 (0)
Nursing home with healthcare	263 (12.4)	232 (16.3)	20 (6.6)	9 (2.6)
Caregiver’s living place in relation to the care receiver, *n* (%)				
Same place	994 (46.8)	432 (30.2)	208 (68)	321 (91.7)
Same building, different place	37 (1.7)	28 (2)	3 (1)	6 (1.7)
Walking distance	339 (16)	294 (20.6)	33 (10.8)	10 (2.9)
30 min commute	410 (19.3)	363 (25.4)	39 (12.7)	5 (1.4)
30 min–1 h commute	217 (10.2)	198 (13.8)	14 (4.6)	4 (1.1)
>1 h commute	128 (6)	115 (8)	9 (2.9)	4 (1.1)
Care receiver’s disease(s)***, *n* (%)				
Dementia	545 (25.3)	336 (23.3)	81 (26.4)	128 (36.2)
Cancer	153 (7.1)	84 (5.8)	22 (7.2)	47 (13.3)
Stroke	186 (8.6)	101 (7)	32 (10.4)	53 (15)
Heart disease	274 (12.7)	165 (11.5)	49 (16)	60 (16.9)
Diabetes	200 (9.3)	110 (7.6)	37 (12.1)	53 (15)
Lung disease	131 (6.1)	73 (5.1)	32 (10.4)	26 (7.3)
Parkinson’s disease	209 (9.7)	114 (7.9)	51 (16.6)	44 (12.4)
Kidney disease	40 (1.9)	22 (1.5)	6 (2)	12 (3.4)
Fractures	134 (6.2)	78 (5.4)	28 (9.1)	28 (7.9)
Physical function limitations	772 (35.8)	504 (35)	129 (42)	139 (39.3)
Sensory limitations	434 (20.1)	312 (21.7)	70 (22.8)	52 (14.7)
Loneliness	430 (19.9)	349 (24.2)	45 (14.7)	36 (10.2)
Psychiatry	257 (11.9)	167 (11.6)	52 (16.9)	38 (10.7)
Substance use	35 (1.6)	22 (1.5)	7 (2.3)	6 (1.7)
Other (such as “old age,” childhood disabilities, autism)	300 (13.9%)	222 (15.4)	42 (13.7)	36 (10.2)
Hours per week of care spent on specific activities, median (IQR)				
Personal help (ADL)	3 (1–12)	1 (0.5–3)	3 (1–7)	12 (3.4–21)
Instrumental help (IADL)	4 (1–14)	2 (1–5)	9 (3.5–16)	21 (8–35)
Supervision	2 (1–7)	1 (0.5–3)	3.5 (1–7)	14 (5–56)
Social support	4 (1.5–14.5)	2 (1–7)	7 (3–21)	28 (10–70)
Medical help	1 (0.5–4)	0.8 (0.3–1.2)	1 (0.5–3.5)	3.5 (1–7)
Arranging services	1.75 (0.5–4)	1 (0.5–2)	2 (1–4)	4 (2–14)

Note: Percentages are adjusted using the sampling weights to produce nationally representative estimates from the sampling frame.

Abbreviations: IQR: interquartile range; ADL: activities of daily living; IADL: instrumental activities of daily living.

* Total *n* = 2102, since 55 caregivers missed the question on intensity of the provided care.

** Housing for older persons (seniorboende) refers to an independent living facility, with minimal service; serviced housing for older persons (serviceboende) refers to a facility where residents are assisted, but do not require regular nursing care; nursing home with healthcare (vård och omsorgboende) refers to municipality-managed homes with healthcare staff.

*** The care receiver can have more than one disease.

**Figure 1.**
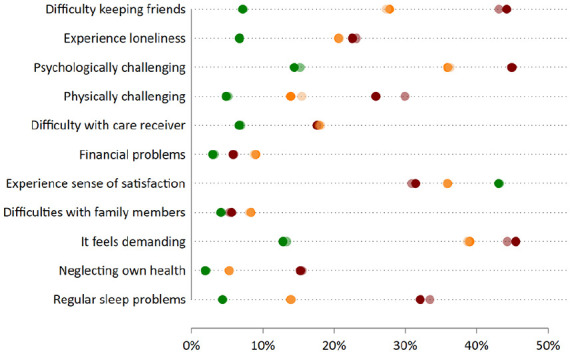
Self-reported experiences of informal caregivers across care intensity levels (*N* = 2157). Dots represent the percentage of informal caregivers reporting this experience often or almost always. Green = low intensity, orange = moderate intensity, red = high intensity. Lighter shades represent crude proportions, while the darker shades are marginal predicted marginal probabilities adjusted for age, sex and level of education.

Higher intensity caregivers reported more negative experiences. Figure 1 presents crude and adjusted estimates of the proportion of low-, moderate-, and high-intensity caregivers that responded “Often” or “Almost always” to the listed disturbances or positive feelings (e.g., sense of satisfaction). The trend of the prevalence of negative experiences increasing across intensity levels was apparent in all items, with some items demonstrating a larger difference between groups than others. For instance, “Difficulty keeping friends,” “Psychologically challenging,” and “Regular sleep problems” were shown to have steep increases according to the intensity of informal caregiving. As expected, the positive item, “Sense of satisfaction” had the opposite direction, with low-intensity caregivers demonstrating a higher sense of satisfaction.

We explored the unmet needs for support services across the care intensity categories. Table II presents the responses to questions about available (offered) support services and the unmet need for services (response option “Wasn’t offered, would like to have it”). For most support services, there was a trend of increasing unmet need across care intensity levels: for instance, only 6.3% of low-intensity caregivers were not offered and would like to have respite care, while this was the case for 17.4% and 26.7% of moderate- and high-intensity caregivers, respectively. Of note, large proportions of caregivers (especially in the low-intensity group) reported not wanting any of the listed services. However, this proportion also declined moving from low- to high-intensity care levels.

**Table II.** Available support and unmet needs of informal caregivers across care intensity levels.

Number of hours of informal care provided per typical week	Yes, I receive it	Was offered, but declined	Wasn’t offered, would like to have it (unmet need)	No, I would not want it
1 to 10 h (*n* = 1441): *n* (%)				
Information and advice	321 (24.2)	28 (2.1)	241 (16.6)	746 (56)
Training and education	57 (4.4)	12 (0.9)	136 (10)	1058 (84.5)
Personal support/counselling	74 (5.6)	23 (1.8)	185 (14.3)	985 (78.2)
Group support/counselling	58 (4.6)	24 (1.9)	112 (8.5)	1060 (84.7)
Feel-good activities	44 (3.4)	18 (1.4)	176 (13.2)	1023 (81.4)
Economic help	57 (4.1)	9 (0.5)	201 (14.9)	1002 (79.9)
Healthcare	89 (6)	8 (0.5)	221 (16.6)	947 (74.8)
Respite care	56 (4.5)	21 (1.7)	87 (6.3)	1104 (87.1)
Technology-based support	38 (2.8)	9 (0.6)	100 (6.9)	1122 (89.5)
Work-related support	19 (1.2)	4 (0.2)	52 (3.3)	1186 (95)
11 to 29 h (*n* = 307), *n* (%)				
Information and advice	97 (33.2)	10 (3.2)	78 (28)	98 (34.6)
Training and education	18 (6.8)	1 (0.3)	58 (21.6)	189 (71)
Personal support/counselling	33 (11.8)	7 (2.6)	75 (27.6)	155 (57.4)
Group support/counselling	16 (5.8)	11 (4)	51 (19.2)	184 (70.6)
Feel-good activities	15 (5.1)	4 (1.4)	72 (26.6)	172 (66.1)
Economic help	20 (7.1)	88 (33.5)	0 (0.0)	159 (58.7)
Healthcare	28 (7.6)	1 (0.3)	84 (31.7)	153 (58.3)
Respite care	31 (11.1)	7 (2.4)	47 (17.4)	184 (68.4)
Technology-based support	14 (4)	2 (0.7)	49 (13.5)	236 (81.2)
Work-related support	12 (2.3)	2 (0.3)	37 (6.9)	215 (90)
30 h or more (*n* = 354), *n* (%)				
Information and advice	126 (38.3)	18 (5.1)	78 (26.1)	96 (29.5)
Training and education	26 (9.1)	7 (1.7)	73 (26.3)	192 (62.4)
Personal support/counselling	34 (10.9)	16 (5.6)	95 (32)	154 (51.1)
Group support/counselling	25 (7.9)	22 (6.4)	71 (24.1)	185 (61.1)
Feel-good activities	20 (5.6)	8 (2.3)	102 (34.5)	165 (56.9)
Economic help	26 (8.8)	2 (0.6)	105 (35.7)	171 (54.3)
Healthcare	45 (11.6)	6 (1.7)	106 (37.1)	135 (46.8)
Respite care	80 (23.9)	20 (5.4)	79 (26.7)	135 (42.9)
Technology-based support	11 (3.4)	6 (1.9)	55 (20)	231 (74.4)
Work-related support	10 (3)	2 (0.5)	24 (8.5)	261 (87.3)

Note: Percentages are adjusted for age, sex, and level of education.

**Figure 2.**
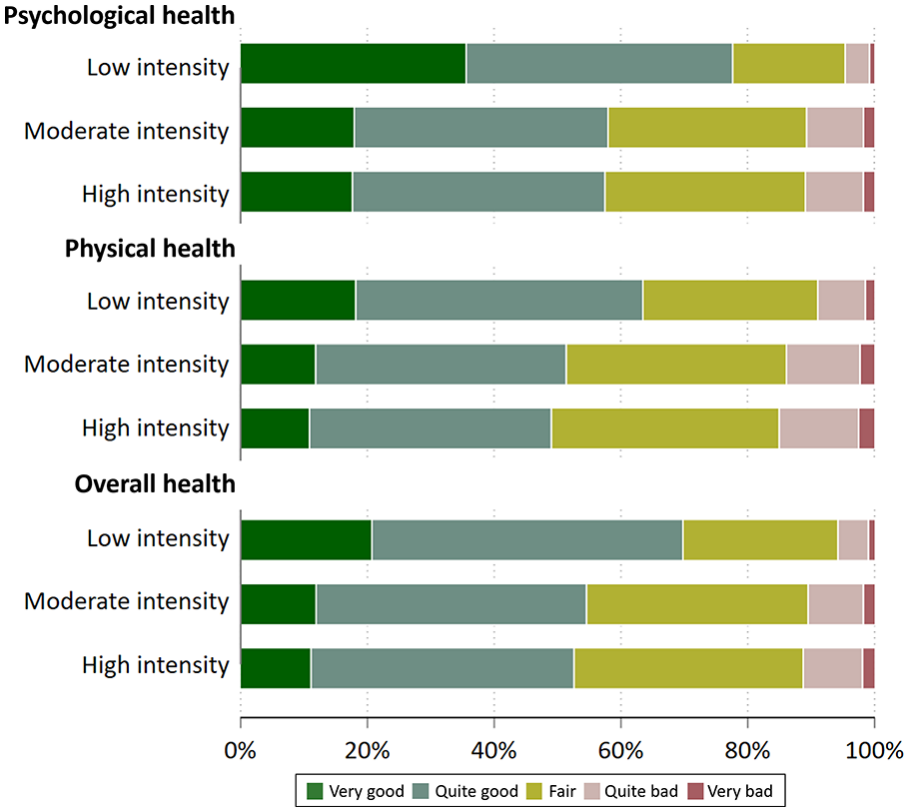
Stacked bar for self-rated physical, psychological, and overall informal caregiver health across care intensity levels (*N* = 2157). Note: Predicted marginal probabilities are reported from logistic regression models adjusted for age, sex, and education level.

Low-intensity caregivers demonstrated better levels of health, especially in the psychological domain, compared to the moderate- and high-intensity groups (Figure 2). While the differences between moderate- and low-intensity caregivers were not as striking, the trend of decreasing self-rated health was present in all domains.

In a sensitivity analysis comparing two questions regarding the intensity of caregiving, we observed some inconsistency between both approaches determining caregiving time, particularly when distinguishing between low- and moderate-intensity groups (Supplementary Figure 2).

## Discussion

Based on a nationally representative survey, this study presents the most up-to-date findings on older informal caregivers in Sweden. While some of our results align with previous Nordic studies—such as the predominance of low-intensity caregiving [13]—our data reveals that almost 1 in 4 (22%) people aged 75+ provides at least 30 h per week of informal care to a relative. More importantly, we identified a strong correlation between the hours spent caregiving and unmet support needs, as well as negative caregiving experiences. These findings reveal that much still needs to be done in Sweden to fully understand, address, and anticipate the challenges faced by older caregivers.

It is not surprising that most older caregivers provide low-intensity care in Sweden, a pattern observed in countries like the Nordics with relatively generous long-term care systems [13]. This can be explained by the phenomenon of *crowding in/crowding out*, whereby generous, formal, long-term care provisions, such as in the Nordics, relieve families from the heavier caregiving tasks, which facilitates lower-intensity, voluntary caregiving to dominate the caregiving landscape [23]. Similarly, according to the 2021 census in England, 6.3% of the adult population provided less intense care, while only 2.8% provided care of 50 or more hours per week [24]. In contrast, Southern European countries, where formal care is less accessible, see higher levels of intensive caregiving, often involving personal care within the same household [25]. In Portugal, 12.4% of older adults (50+) were providing intrahousehold care in 2010–2011, compared to just 4% in Sweden and 4.5% in Denmark [26]. A similar trend is seen in the United States, where 32% of caregivers provide high-intensity care (21+ h per week), and 52% of low-intensity caregivers report having “no choice” in their role [27]. Most studies impose upper age limits, typically around 75–80 years of age, making it difficult to compare our findings, which included caregivers aged 65 and older, with a significant number in their eighties and nineties. While cross-country comparisons can be challenging [28], the clear relationship between welfare state generosity and the predominance of low-intensity caregiving remains consistent in our up-to-date findings.

It has been shown that high-intensity caregiving is detrimental, with numerous studies linking it to stress, health decline, and unmet support needs [29–31]. However, our study offers a new and crucial perspective by highlighting these effects in a largely under-researched group—older caregivers. While most research has focused on younger caregivers or combined age groups, our findings specifically spotlight those over 65, revealing that they too face significant negative consequences from high-intensity caregiving. Additionally, we show that older women and spousal caregivers are disproportionately represented in these high-intensity groups, reinforcing patterns seen in the broader caregiving literature [8,24,31]. This highlights that a gender care gap still persists in Sweden, despite an encouraging trend toward narrowing it [32]. There might be several explanations as to why higher intensity caregiving is associated with more negative consequences. Some reasons are quite straightforward: because individuals are occupied for more hours each day, they may lack the time to maintain their social connections, go for walks, or engage in leisure activities. Moreover, being an informal caregiver has been associated with higher odds of refraining from seeking medical care and with medication non-adherence [33], and this mechanism could be particularly relevant for the older caregivers, who themselves experience increasing healthcare needs due to the health decline inherent in aging. In addition, caregiving might be linked to chronic stress, either due to a stressful daily schedule and sleep disturbances (i.e., *caregiver effect* [34]) and/or to being worried about a loved one (i.e., *family effect* [35]).

One of the most policy-relevant findings of our survey is the clear pattern of increasing unmet needs for support services as caregiving intensity rises. High-intensity caregivers, 64% of whom are over 75, face significantly greater unmet needs for services like respite care compared to low-intensity caregivers. This is likely due to their increasing care needs as they age, requiring more support to continue in their caregiving role. Given the current political discourse on social care in Sweden, namely the forthcoming adoption of a new Social Services Act focusing on preventive and proactive social services [36], these findings are crucial for informing future reforms. A more detailed investigation at the municipal level is needed to better understand and address the needs of all caregivers, particularly older and more vulnerable groups, to ensure they receive the necessary support.

### Strengths and limitations

The study is based on a large-scale population survey with a sample drawn to be representative of the population. Thus, we comprehensively captured the reality of older informal caregivers in Sweden, an often-overlooked demographic. However, this study comes with several limitations. First and foremost, in interpreting the findings, it is important to avoid making direct comparisons between surveys, as definitions of care, sample characteristics, response rates, and the method of ascertaining intensity in different studies vary significantly. For example, a study by Tur Sinai et al. compared the prevalence of older caregivers (aged 50 and above) across three surveys: the Survey of Health, Ageing and Retirement in Europe (data from 2015), the European Health Interview Survey (data from 2014), and the European Quality of Life Survey (data from 2016). The prevalence of informal caregiving in Sweden was 25.14%, 10.76%, and 20%, respectively [2].

Second, measuring care intensity presents several challenges. In our survey, we measured intensity in two ways: a categorical question and a task-specific question. The latter relies on respondents’ mathematical reasoning and may not reliably capture the overall intensity of care provided, as shown in our sensitivity analysis. Therefore, we used the former simpler question on the total number of caregiving hours per week. Additionally, there is concern that care intensity might be underreported in surveys. Rutherford et al. used data from the English Longitudinal Study of Ageing to compare caregivers’ and care receivers’ responses to different questions on informal care. They concluded that standard measures are likely to underestimate both the scale and scope of informal care [37]. However, it is unclear whether the underreporting applies to all population contexts.

Finally, despite a higher response rate compared to previous surveys, we do not have information about the caregiving status of non-responders in the survey, allowing for non-responder bias. On the one hand, it could be hypothesized that more burdened, busy caregivers are less likely to respond when invited to complete surveys. For instance, in an Australian cohort study, being a full-time caregiver was associated with significantly decreased ongoing participation in the study [38]. On the other hand, people are more likely to participate in research when the topic is relevant to their life situation [39]. Indeed, another study comparing responder and non-responder caregivers found that caregivers who responded to additional questionnaires provided more burdensome, often spousal care, compared to non-responders [40].

### Implications for research and practice

Our findings provide further evidence that intensity of caregiving is an important axis for stratifying caregivers and examining their experiences, also in old age. Older people, often seen as *receivers* of informal care, have not traditionally been prioritized in caregiving research, and it is crucial to include them in future studies. Considering a growing recognition of the role played by informal caregivers within long-term care (LTC), as witnessed by the 2022 European Care Strategy [41], and the 2023 partnership agreement between the World Health Organization and the European Commission for better LTC [42], we derive several recommendations for policy makers and decision makers. First, it is imperative for health and care professionals to take steps to proactively identify and support informal caregivers, and even more importantly older informal caregivers given their greater risk of negative health outcomes. One potential avenue for identification could be through primary care services, which are often the first point of contact for individuals seeking healthcare assistance. Second, tailoring support interventions to meet older caregivers’ needs is essential, rather than adopting a one-size-fits-all approach. Factors such as the intensity of provided informal care may determine the need for (and type of) support services. Third, establishing a comprehensive quality register of informal caregivers would enable ongoing monitoring of their health and well-being, ensuring they receive the necessary support and resources on a more proactive basis. Additionally, it is essential to incorporate the perspective of informal caregivers, and more specifically older caregivers, in formal care reforms, reflecting the situations of both high-intensity care and low-intensity caregivers. By integrating their insights into policy development and service provision, we may foster a more inclusive and responsive healthcare system that acknowledges and addresses the needs of both informal caregivers and care receivers.

## Conclusion

This study provides up-to-date evidence on the landscape of informal caregiving by older people in Sweden. Our findings revealed distinct patterns in caregiving intensity and demographic characteristics, with most older caregivers providing several hours of care each week, while a significant minority engage in high-intensity caregiving. Notably, low-intensity caregivers demonstrated better psychological health than their moderate- and high-intensity counterparts, highlighting the crucial link between caregiving intensity and health outcomes. Of note, as caregiving demands increased, so did negative experiences and unmet support needs. These results emphasize the urgent need for tailored support interventions that address the diverse and evolving needs of older informal caregivers across the intensity spectrum, ensuring their well-being and sustainability in this vital role.

## Supplemental Material

sj-docx-1-sjp-10.1177_14034948251335113 – Supplemental material for The intensity of informal caregiving and its implications for older caregivers: a national survey in Sweden Supplemental material, sj-docx-1-sjp-10.1177_14034948251335113 for The intensity of informal caregiving and its implications for older caregivers: a national survey in Sweden by Mariam Kirvalidze, Elizabeth Hanson, Lennart Magnusson, Lena Dahlberg, Anders Wimo, Lucas Morin and Amaia Calderón-Larrañaga in Scandinavian Journal of Public Health

sj-pdf-2-sjp-10.1177_14034948251335113 – Supplemental material for The intensity of informal caregiving and its implications for older caregivers: a national survey in Sweden Supplemental material, sj-pdf-2-sjp-10.1177_14034948251335113 for The intensity of informal caregiving and its implications for older caregivers: a national survey in Sweden by Mariam Kirvalidze, Elizabeth Hanson, Lennart Magnusson, Lena Dahlberg, Anders Wimo, Lucas Morin and Amaia Calderón-Larrañaga in Scandinavian Journal of Public Health
