# Supplementary material for: The intensity of informal caregiving and its implications for older caregivers: a national survey in Sweden
Source: Scand J Public Health. 2025 May 1;54(5):512–21. doi: 10.1177/14034948251335113 (PMC13323912; doi:10.1177/14034948251335113)

**Supplementary materials**

**Supplementary Table 1.** Distribution of responders and nonresponders in terms of age, sex, education and birthplace (row percentages).

|  | **Responded** | **Did not respond** |
| --- | --- | --- |
| **No (% of sampling frame)** | 16959 (54.9%) | 13935 (45.1%) |
| **Sex, No (%)** |  |  |
| Male | 7880 (53.8%) | 6756 (46.2%) |
| Female | 9079 (55.8%) | 7179 (44.2%) |
| **Age, No (%)** |  |  |
| 55-64 years old | 2159 (42.2%) | 2959 (57.8%) |
| 65-74 years old | 7512 (58.8%) | 5263 (41.2%) |
| 75-84 years old | 5826 (60.4%) | 3823 (39.6%) |
| >84 years old | 1462 (43.6%) | 1890 (56.4%) |
| **Birthplace, No (%)** |  |  |
| Born outside Sweden | 1733 (37.8%) | 2856 (62.2%) |
| Born in Sweden | 15226 (57.9%) | 11079 (42.1%) |
| **Highest educational attainment, No (%)** |  |  |
| High education | 6144 (65.7%) | 3211 (34.3%) |
| Medium education | 2321 (59.6%) | 1571 (40.4%) |
| Low education | 8494 (48.1%) | 9153 (51.9%) |

**Supplementary Table 2.** Checklist of reporting items for PRICSSA guidelines.

| **Item** | **Description** | **Page (section)** |
| --- | --- | --- |
| Descriptive items | | |
| 1.1 Data collection dates | Describe the survey’s data collection dates (e.g., range) to provide historical context that could affect survey responses and nonresponse. | **Methods** |
| 1.2 Data collection mode(s) | Describe the survey’s data collection mode(s). Data collection mode can affect survey responses (e.g., to sensitive questions), including nonresponse, and a survey’s data collection mode may change over time (e.g., during the COVID-19 pandemic). | **Methods** |
| 1.3 Target population | State the target population the survey was designed to represent and describe all weighted estimates with respect to this target population. | **Methods** |
| 1.4 Sample design | Describe the survey’s sample design, including information about stratification, cluster sampling, and unequal probabilities of selection. | **Methods** |
| 1.5 Survey response rate(s) | State the survey’s response rate and how it was calculated. | **Results**  **Supplementary Table 2** |
| Analytical items | | |
| 2.1 Missingness rates | Report rates of missingness for variables of interest and models and describe any methods (if any) for dealing with missing data (e.g., multiple imputation). | **Methods** |
| 2.2 Observation deletion | State whether any observations were deleted from the dataset. If observations were deleted, provide a justification. Note: It is best practice to avoid deleting cases and use available subpopulation analysis commands no matter what variance estimation method is used. | **Methods** |
| 2.3 Sample sizes | Include unweighted sample sizes for all weighted estimates. | **NA** |
| 2.4 Confidence intervals/  standard errors | Include confidence intervals or standard errors when reporting all estimates to inform the reliability/precision of each estimate. | **Results, as applicable** |
| 2.5 Weighting | State which analyses were weighted and specify which weight variables were used in analysis. | **Methods**  **Table 1** |
| 2.6 Variance estimation | Describe the variance estimation method used in the analysis and specify which design variables (e.g., PSU/stratum, replicate weights) were used. | **NA** |
| 2.7 Subpopulation analysis | Describe the procedures used for conducting subpopulation analyses (e.g., Stata’s “subpop” command, SAS’s “domain” command). | **NA** |
| 2.8 Suppression rules | State whether or not a suppression rule was followed (e.g., minimum sample size or relative standard error). | **NA** |
| 2.9 Software and code | Report which statistical software was used, comprehensively describe data management and analysis in the manuscript, and provide all statistical software code. | **Methods** |
| 2.10 Singleton problem (as needed) | Taylor Series Linearization requires at least two PSUs per stratum for variance estimation. Sometimes an analysis is being performed and there is only a single PSU in a stratum. There are several possible fixes to this problem, which should be detailed if the singleton problem is encountered. | **NA** |
| 2.11 Public/restricted data (as needed) | If applicable, state whether the public use or restricted version of the dataset was analyzed. | **NA** |
| 2.12 Embedded experiments (as needed) | If applicable, provide information about split sample embedded experiments (e.g., mode of data collection or varying participant incentives) and detail whether experimental factors were accounted for in the analyses. | **NA** |

**Supplementary Table 3.** Number and proportion of caregivers across characteristics of the entire study sample (N=16,959).

|  | **No (% of total sample*)** |
| --- | --- |
| **Total** | 2585 (16.8%) |
| **Sex** |  |
| Male | 1050 (14.5%) |
| Female | 1535 (18.8%) |
| **Age, years** |  |
| 55–65 | 428 (22.4%) |
| 65–75 | 1086 (16.8%) |
| 75–85 | 830 (12.4%) |
| >85 | 241 (11.4%) |
| **Birthplace** |  |
| Born outside Sweden | 279 (16.3%) |
| Born in Sweden | 2306 (16.9%) |
| **Highest educational attainment** |  |
| Lower than high school | 395 (12.8%) |
| High school | 1144 (17.1%) |
| Higher education | 1039 (18.7%) |
| **Retirement status** |  |
| Fully retired | 1841 (13.8%) |
| Working | 388 (21.9%) |
| Retired, but working part-time | 190 (15.8%) |
| Working, on sick leave | 18 (23%) |
| Voluntarily not working | 27 (29.2%) |
| **Civil status** |  |
| Married or partnered | 1667 (19%) |
| Not married | 355 (18%) |
| Divorced | 396 (15.3%) |
| Widow | 167 (7.4%) |
| **Quartiles of disposable income per household** |  |
| Lowest (up to 250 thousand SEK) | 930 (14.7%) |
| 2nd (251-415 thousand SEK) | 1007 (18.6%) |
| 3rd (416-669 thousand SEK) | 480 (17.4%) |
| Highest (670+ thousand SEK) | 168 (15.6%) |

**Percentages are calculated using sampling weights to recreate the Swedish population*

**Supplementary Figure 1.** Sample selection flowchart.


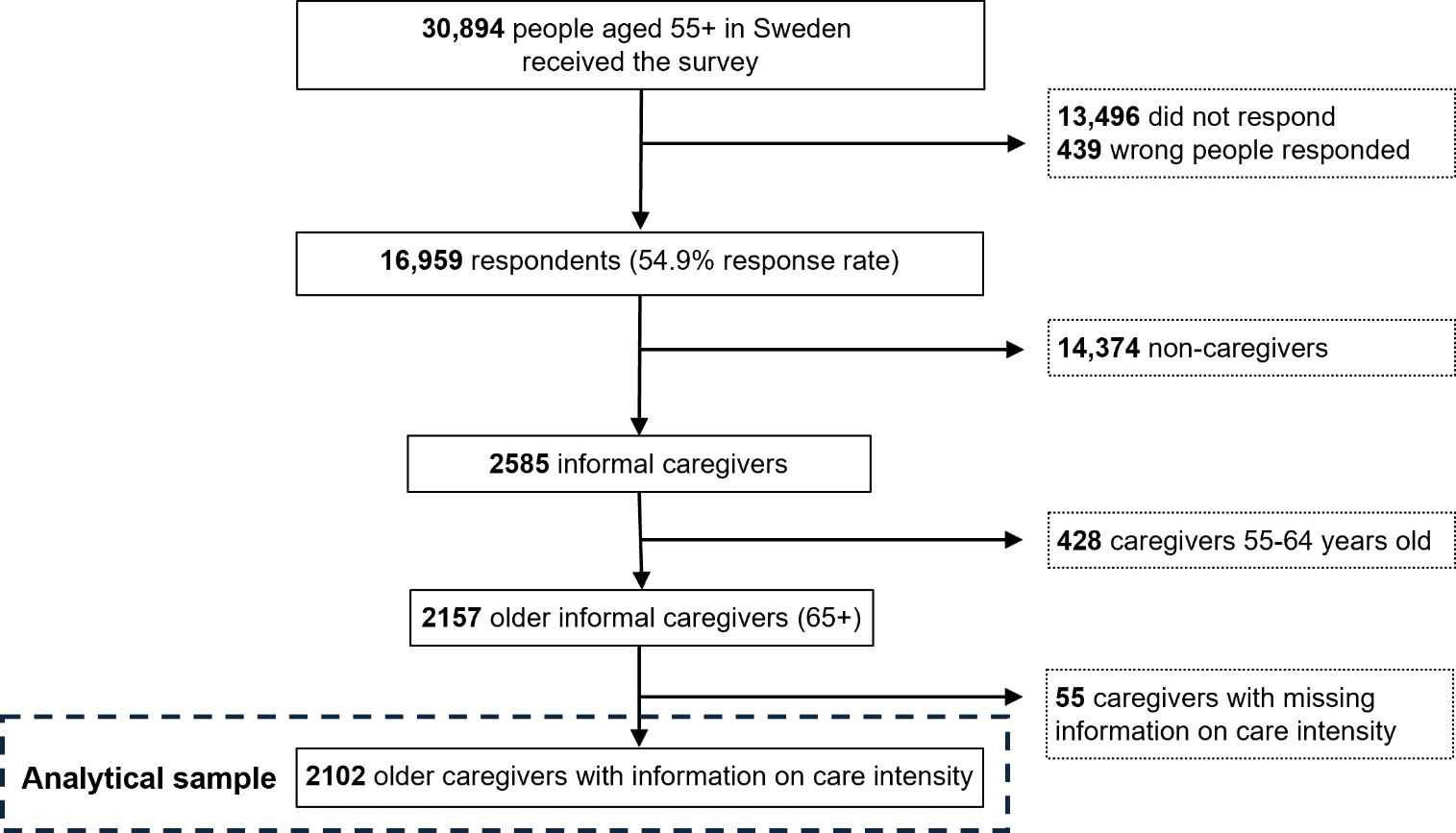


**Supplementary Figure 2.** Sensitivity analysis comparing two questions related to intensity of care.

|  | | | | | **Percentiles** | | |
| --- | --- | --- | --- | --- | --- | --- | --- |
|  | **Intensity** | **N** | **Minimum** | **Maximum** | **25th** | **50th** | **75th** |
| **Hours per week** | Low | 1441 | 0 | 168 | 1.83 | 4.02 | 10.50 |
|  | Moderate | 307 | 0 | 168 | 9.00 | 21.00 | 42.00 |
|  | High | 354 | 0 | 168 | 28.12 | 70.00 | 147.00 |


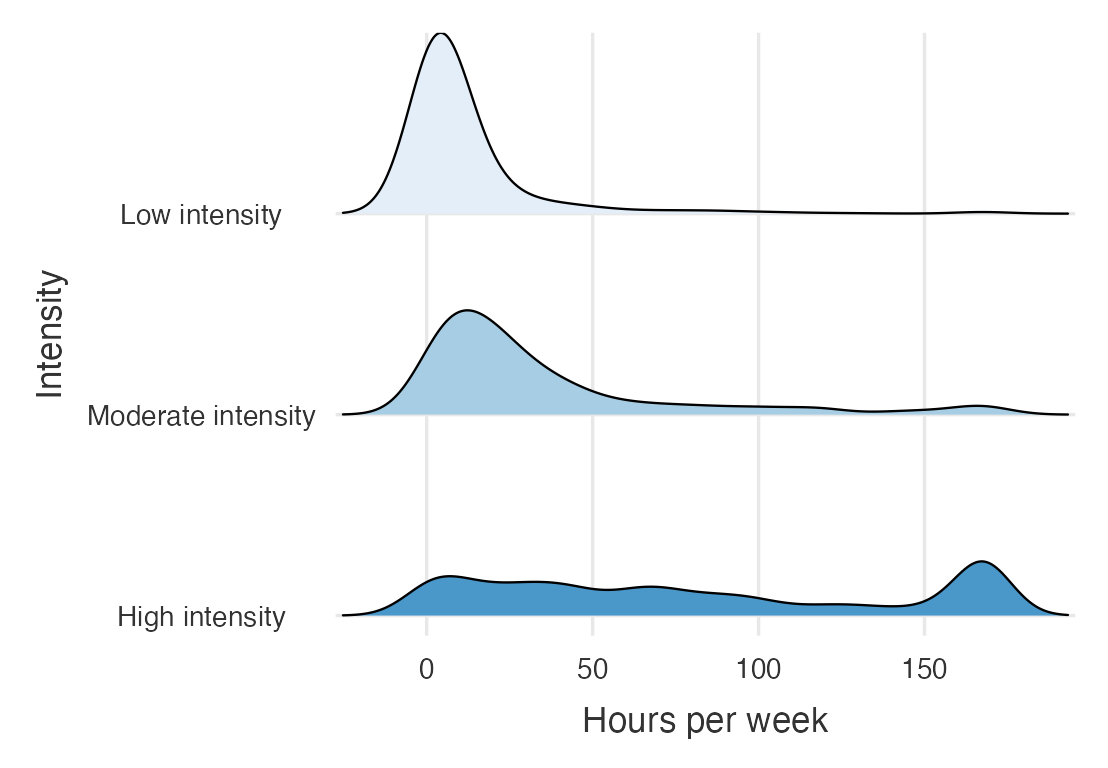


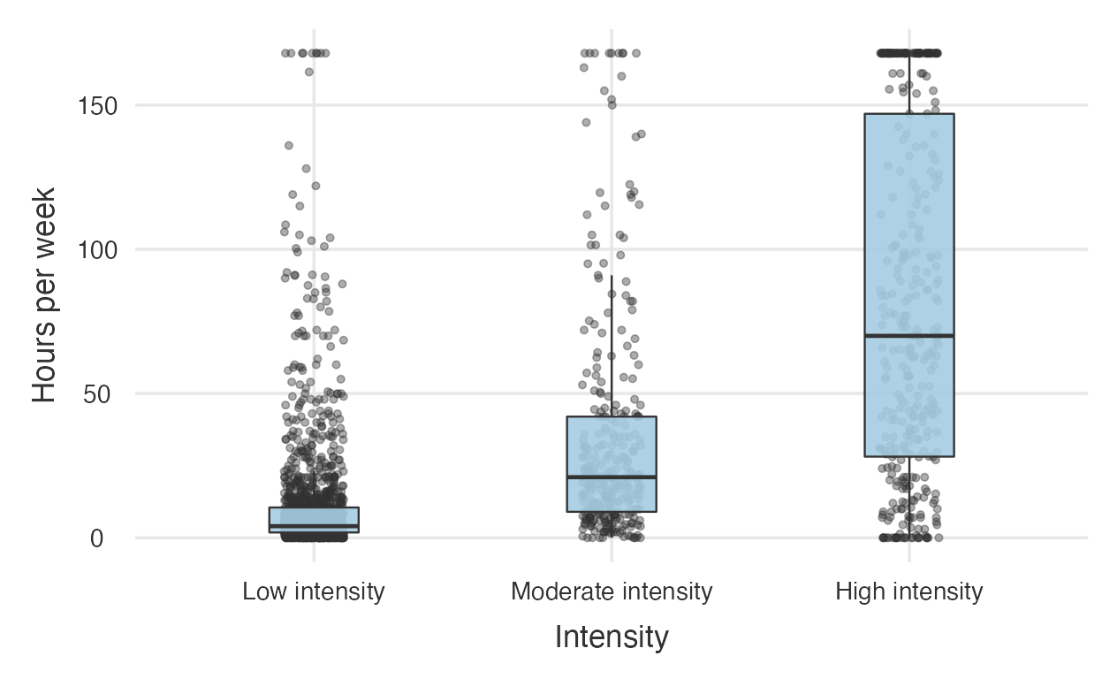

Supplement: sj-docx-1-sjp-10.1177_14034948251335113 – Supplemental material for The intensity of informal caregiving and its implications for older caregivers: a national survey in Sweden [file sj-docx-1-sjp-10.1177_14034948251335113.docx]
