# Supplementary material for: The intensity of informal caregiving and its implications for older caregivers: a national survey in Sweden
Source: Scand J Public Health. 2025 May 1;54(5):512–21. doi: 10.1177/14034948251335113 (PMC13323912; doi:10.1177/14034948251335113)
Supplement: sj-pdf-2-sjp-10.1177_14034948251335113 – Supplemental material for The intensity of informal caregiving and its implications for older caregivers: a national survey in Sweden [file sj-pdf-2-sjp-10.1177_14034948251335113.pdf]

Vårdar, hjälper eller stödjer du en närstående?

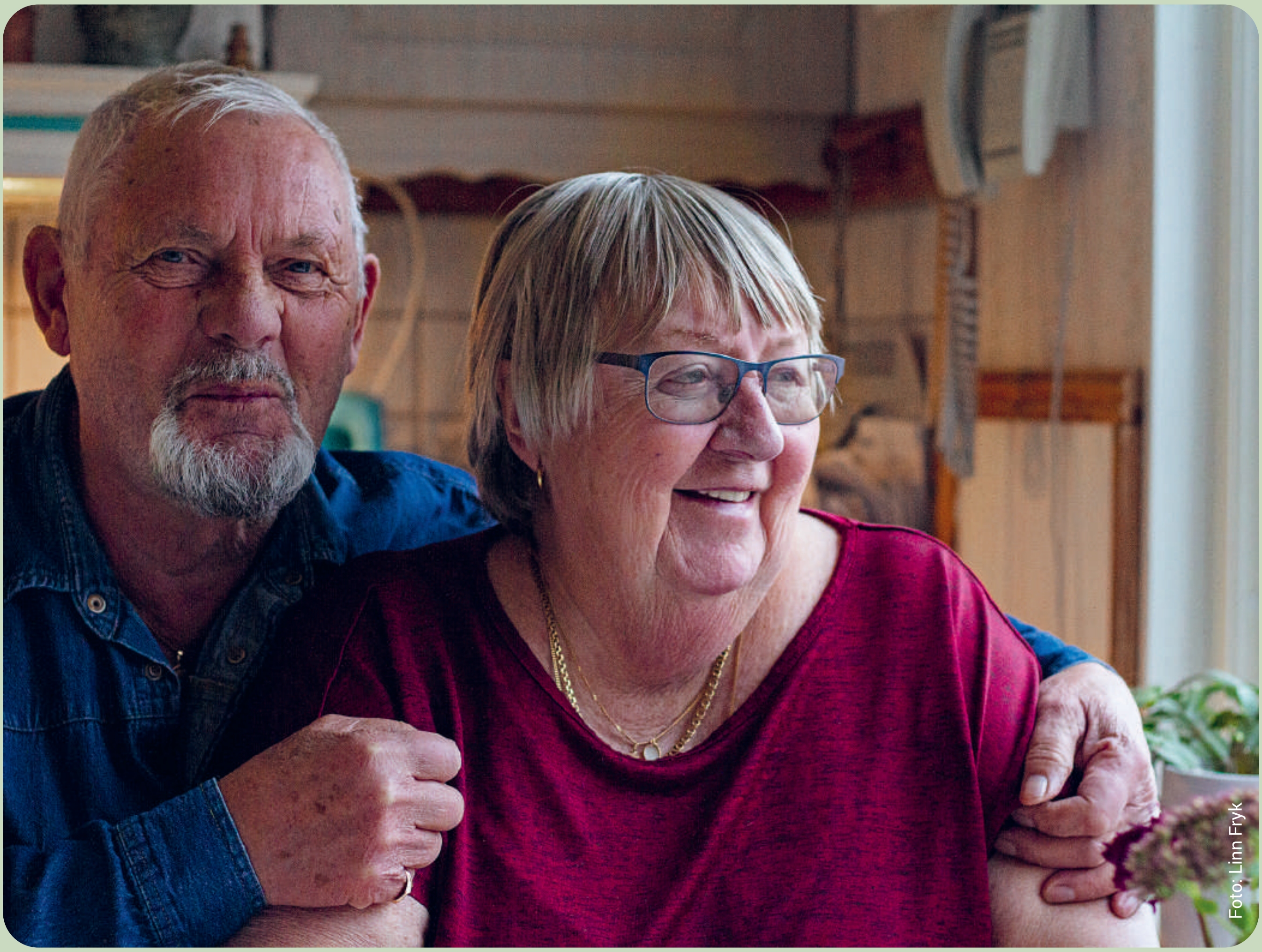

SCB

Vård, hjälp eller stöd till en närstående

Introduktion och bakgrund till undersökningen

Vad är syftet med denna undersökning?

Den här undersökningen ska ge en bild av dom som regelbundet ger vård, hjälp eller stöd till en närstående på grund av fysisk eller psykisk sjukdom, funktionsnedsättning eller ålderdom.

Jag ger inte vård, hjälp eller stöd: ska jag svara på enkäten?

Ja, dina svar är viktiga även om du inte ger vård eller stöd till en närstående, men det är då bara några få frågor vilket tar cirka 3 minuter.

Vad menar vi med “närstående”?

Med närstående menar vi någon i din familj, släkt eller någon annan som du har en nära relation till såsom vän, granne eller arbetskamrat.

Vad menar vi med “vård, hjälp eller stöd”?

Med vård, hjälp eller stöd menar vi sådant som du gör någorlunda regelbundet till någon med funktionsnedsättning, långvarig sjukdom eller andra behov. Du kanske vårdar, hjälper eller stödjer din partner, ett barn, andra familjemedlemmar, släktingar eller närstående. Tänk på all typ av vård, hjälp och stöd som du ger någon, utöver den vanliga omsorgen om barn utan särskilda behov. Det kan vara personlig omsorg eller tillsyn. Det kan vara mer praktiska sysslor som inköp, att skjutsa någon, skötsel av hus och hem, eller hjälp i kontakten med myndigheter eller vårdpersonal. Det kan också vara hjälp med att sköta ekonomi, post, räkningar och liknande eller att bryta social isolering. Detta är bara några exempel på att ge vård, hjälp eller stöd.

I frågorna nedan använder vi orden "vård eller stöd" som ett samlingsnamn för alla former av vård, hjälp eller stöd som du ger.

Vilken typ av hjälp eller stöd ingår inte?

Den här undersökningen handlar inte om den hjälp, vård eller stöd som du ger i ditt yrke t.ex. som vård- och omsorgspersonal, som personlig assistent eller som god man. Undersökningen handlar endast om vård, hjälp och stöd som du ger som privatperson. Undersökningen handlar inte om den vård som människor ger till barn som inte har särskilda behov.

OBS! Vi ber att bara du som personligen fått det här brevet svarar på enkäten och bara svarar för dig själv.

Genom att svara på denna enkät ger du samtycke till att den tillhandahållna informationen används för forskningsändamål av forskare vid Aging Research Center, Karolinska Institutet och Nationellt kompetenscentrum anhöriga. Dina uppgifter kommer att avpersonifieras och hanteras konfidentiellt, och ditt namn kommer inte att synas i forskningspublikationer eller rapporter.

Frågeblanketten läses maskinellt. Vi ber dig därför att:

- Använda bläckpenna
- Skriva tydliga siffror, så här: 1 2 3 4 5 6 7 8 9 0
- Markera dina svar med kryss, så här: ☒
- Om du svarat fel täck hela rutan med det felaktiga krysset, så här: ■

1. Vilket år är du född?
Skriv årtalet med fyra siffror, exempelvis 1986 eller 2001.

Födelseår: ☐☐☐☐

2. Är du man, kvinna eller har annan könsidentitet?

☐ Man
☐ Kvinna
☐ Annat

3. Vilken är din nuvarande huvudsakliga sysselsättning?
Endast ett alternativ kan anges. Ange det alternativ som stämmer bäst för dig.

☐ Jag är pensionerad
☐ Jag arbetar ☐☐☐ % av heltid
☐ Jag är pensionerad, men jag arbetar ☐☐☐ % av heltid
☐ Jag arbetar, men är nu sjukskriven ☐☐☐ % av heltid
☐ Jag är hemmafru/man (inte pensionär)
☐ Annat, skriv i rutan: ____________________

4. Vårdar eller stödjer du regelbundet (dvs. inte bara tillfälligt under en kortare period) någon eller några närstående personer?
Med vård eller stöd menar vi vardagliga sysslor, personlig omsorg eller annat stöd som behövs på grund av fysisk eller psykisk sjukdom, funktionsnedsättning eller ålder. Läs gärna introduktionen igen om du är osäker på om du tillhör den här gruppen!

☐ Ja, en person
☐ Ja, två personer
☐ Ja, tre personer
☐ Ja, mer än tre personer
☐ Nej → *Gå till fråga 25*

5. Hur ofta ger du vård eller stöd till någon närstående person (till en eller flera personer)?

- ☐ Varje dag
- ☐ Minst en gång i veckan
- ☐ Minst en gång i månaden
- ☐ Mer sällan än en gång i månaden → *Gå till fråga 25*

6. Ungefär hur många timmar i genomsnitt per vecka ger du vård eller stöd till någon närstående person (till en eller flera personer sammanlagt)?

- ☐ Mindre än 1 timme per vecka
- ☐ 1–10 timmar per vecka
- ☐ 11–29 timmar per vecka
- ☐ 30–59 timmar per vecka
- ☐ 60 timmar eller mer per vecka

Nu kommer några frågor om den person som du ger vård eller stöd. Om du ger vård eller stöd till fler än en person ber vi dig tänka på den person du ger mest vård eller stöd.

7. Vem är den du vårdar eller stödjer mest?

- ☐ Make/maka/partner
- ☐ Barn
- ☐ Förälder
- ☐ Syskon
- ☐ Släkting
- ☐ God vän, granne, bekant

8. Ger du vård eller stöd till mer än en person?
Du kan markera flera alternativ.

- ☐ Nej, jag ger bara stöd till en person som angavs i föregående fråga
- ☐ Ja, make/maka/partner
- ☐ Ja, barn
- ☐ Ja, förälder
- ☐ Ja, syskon
- ☐ Ja, släkting
- ☐ Ja, god vän, granne, bekant

9. Vilket kön har den person som du ger mest vård eller stöd?

- ☐ Man
- ☐ Kvinna
- ☐ Annat

10. Talar den person som du ger mest vård eller stöd svenska?

- ☐ Ja, flytande
- ☐ Ja, delvis
- ☐ Nej, inte alls

11. Hur gammal är den person som du ger mest vård eller stöd?
Om du är osäker så gör en uppskattning.

☐☐☐ år

Om du är osäker på ålder kan du också ange födelseår.

Född år: ☐☐☐☐

12. Vilken boendeform har den person som du ger mest vård eller stöd?

- ☐ Ordinärt boende (villa eller lägenhet)
- ☐ Trygghetsboende, seniorboende
- ☐ Serviceboende, servicebostad
- ☐ Särskilt boende, gruppboende, vård- och omsorgsboende

13. Var bor du i relation till den person som du ger mest vård eller stöd?

- ☐ I samma hushåll
- ☐ I olika hushåll men samma byggnad
- ☐ Inom gångavstånd
- ☐ Inte inom gångavstånd, men inom 30 minuters enkel resa
- ☐ Mellan 30 minuter och 1 timmes enkel resa
- ☐ Mer än 1 timmes enkel resa

14. Vilken är den främsta anledningen till att den person som du ger mest vård eller stöd behöver din hjälp?
Endast ett alternativ kan anges. Om personen har mer än ett hälsoproblem kan du ange detta i fråga 15.

- ☐ Alzheimers sjukdom, demens, minnesproblem
- ☐ Cancer
- ☐ Stroke
- ☐ Hjärtinfarkt, hjärtsvikt eller annan hjärtsjukdom
- ☐ Diabetes
- ☐ Lungproblem (KOL, astma)
- ☐ Parkinsons sjukdom eller andra neurologiska problem (t.ex. epilepsi)
- ☐ Njursjukdom (t.ex. dialys)
- ☐ Fraktur (t.ex. höftfraktur efter ett fall)
- ☐ Fysisk funktionsnedsättning
- ☐ Problem med syn eller hörsel
- ☐ Ensamhet, social isolering
- ☐ Psykiska hälsoproblem (t.ex. depression, schizofreni)
- ☐ Missbruk av alkohol eller droger
- ☐ Andra skäl, förklara med egna ord:

15. Finns det ytterligare anledningar till att den person som du ger <u>mest</u> vård eller stöd behöver din hjälp?
Du kan markera flera alternativ.

- ☐ Nej, det finns inga ytterligare anledningar

- ☐ Alzheimers sjukdom, demens, minnesproblem
- ☐ Cancer
- ☐ Stroke
- ☐ Hjärtinfarkt, hjärtsvikt eller annan hjärtsjukdom
- ☐ Diabetes
- ☐ Lungproblem (KOL, astma)
- ☐ Parkinsons sjukdom eller andra neurologiska problem (t.ex. epilepsi)
- ☐ Njursjukdom (t.ex. dialys)
- ☐ Fraktur (t.ex. höftfraktur efter ett fall)
- ☐ Fysisk funktionsnedsättning
- ☐ Problem med syn eller hörsel
- ☐ Ensamhet, social isolering
- ☐ Psykiska hälsoproblem (t.ex. depression, schizofreni)
- ☐ Missbruk av alkohol eller droger
- ☐ Andra skäl, förklara med egna ord:

Nu kommer några frågor om den vård eller det stöd du ger.

16. Om du tänker på en <u>typisk dag</u> när du ger vård eller stöd (till en eller flera personer), ungefär hur mycket tid ägnar du då åt följande uppgifter?

	Timmar	Minuter	Inte aktuellt
Hjälp med toalettbesök, att äta, påklädning, personvård, att gå och att duscha/bada			☐
Handla mat, matlagning, hushållssysslor, tvätt, transport, medicinering och hantering av ekonomin			☐
Tillsyn (dvs. se till, påminna, motivera, stödja och förebygga faror)			☐
Umgänge, ge socialt eller känslomässigt stöd			☐
Medicinsk hjälp (t.ex. sårvård, injektioner, hjälp med rehabilitering)			☐
Samordna vården, kontakta hälso- och sjukvården och/eller socialtjänsten (äldreomsorgen)			☐

17. Om du tänker på en <u>typisk vecka</u> när du ger vård eller stöd (till en eller flera personer), ungefär hur många dagar ägnar du då åt följande uppgifter?

	Dagar per vecka	Inte aktuellt
Hjälp med toalettbesök, att äta, påklädning, personvård, att gå och att duscha/bada	☐	☐
Handla mat, matlagning, hushållssysslor, tvätt, transport, medicinering och hantering av ekonomin	☐	☐
Tillsyn (dvs. se till, påminna, motivera, stödja och förebygga faror)	☐	☐
Umgänge, ge socialt eller känslomässigt stöd	☐	☐
Medicinsk hjälp (t.ex. sårvård, injektioner, hjälp med rehabilitering)	☐	☐
Samordna vården, kontakta hälso- och sjukvården och/eller socialtjänsten (äldreomsorgen)	☐	☐

18. Är det någon annan förutom du som vårdar eller stödjer personen du hjälper <u>mest</u>?
Du kan markera flera alternativ.

☐ Ja, familjemedlemmar, släktingar och/eller vänner
☐ Ja, äldreomsorg såsom hemtjänst, dagverksamhet, särskilt boende e.d. (från kommunen eller ett privat företag)
☐ Ja, hälso- och sjukvård, hemsjukvård
☐ Ja, väntjänst eller annan verksamhet av frivilligorganisationer
☐ Ja, privatköpt hjälp (t.ex. städservice)

☐ Nej

19. a. Hur länge har du gett vård eller stöd till den person du för närvarande hjälper <u>mest</u>?

☐ 0–3 månader
☐ 4–6 månader
☐ 7 månader–1 år
☐ 2–5 år
☐ 6–10 år
☐ Mer än 10 år

b. Hur länge har du gett vård eller stöd till någon annan dessförinnan (ange den totala tiden om du har vårdat flera personer)?

☐ Jag har inte vårdat någon annan än den här personen
☐ 0–3 månader
☐ 4–6 månader
☐ 7 månader–1 år
☐ 2–5 år
☐ 6–10 år
☐ Mer än 10 år

Nu kommer några frågor om olika typer av stöd som du kan behöva eller få. Dessa frågor avser total vård du ger (till en eller flera personer).

20. Har du fått och/eller erbjudits stöd från din kommun eller andra organisationer i form av...
Markera ett svar för varje fråga.

	Ja	Ja, blivit erbjuden men avböjt	Nej, men skulle vilja ha det	Nej, och är inte intresserad
...information och rådgivning?	☐	☐	☐	☐
...utbildning?	☐	☐	☐	☐
...personligt stödsamtal?	☐	☐	☐	☐
...stödgrupp?	☐	☐	☐	☐
...må bra aktiviteter (t.ex. stavgång, vattengymnastik, massage)?	☐	☐	☐	☐
...ekonomiskt bidrag eller ekonomiskt stöd?	☐	☐	☐	☐
...hälsoundersökning och hälsorådgivning?	☐	☐	☐	☐
...avlösning (i hemmet eller via dagverksamhet, korttids- eller växelvård)?	☐	☐	☐	☐
...stöd via modern teknik eller Internet (t.ex. webbinformation, videokonferens, grupper på Internet, GPS-system)?	☐	☐	☐	☐
...stöd som underlättar arbetet (möjlighet till distansarbete, flexibel arbetstid, kommunicera med arbetsledare via Internet eller liknande)?	☐	☐	☐	☐

21. Känner du personligen att du själv som anhörigvårdare får tillräckligt med stöd från…

	Ja, absolut	Ja, för det mesta	Nej	Behöver inte/vill inte
…äldreomsorgen?	☐	☐	☐	☐
…hälso- och sjukvården?	☐	☐	☐	☐
…frivilligorganisationer?	☐	☐	☐	☐
…familj/vänner/grannar?	☐	☐	☐	☐

Nu kommer några frågor om din situation som anhörigvårdare, dina känslor och din hälsa. Dessa frågor avser total vård du ger (till en eller flera personer).

22. Under den senaste månaden, hur mycket eller lite skulle du uppskatta att din sömn störs på grund av den vård eller stöd du ger?

- ☐ Min sömn störs inte alls
- ☐ Ungefär 1 natt per vecka
- ☐ Mellan 2 och 3 nätter per vecka
- ☐ Mellan 4 och 6 nätter per vecka
- ☐ Varje natt

23. Om du tänker på den vård och det stöd du sammantaget ger, hur ofta innebär det att…

	Alltid/nästan alltid	Ofta	Ibland	Sällan/aldrig	Vet inte/ vill inte svara
...du har svårt att hinna umgås med dina vänner?	☐	☐	☐	☐	☐
...du upplever ensamhet?	☐	☐	☐	☐	☐
...du har svårt att hinna motionera, exempelvis att ta en promenad eller gå till gymmet?	☐	☐	☐	☐	☐
...det är psykiskt påfrestande för dig?	☐	☐	☐	☐	☐
...det är fysiskt påfrestande för dig?	☐	☐	☐	☐	☐
...du upplever problem i din relation till den/de person(er) som du ger vård eller stöd?	☐	☐	☐	☐	☐
...du får ekonomiska problem?	☐	☐	☐	☐	☐
...du upplever en känsla av tillfredsställelse?	☐	☐	☐	☐	☐
...du upplever problem i dina relationer med familjemedlemmar?	☐	☐	☐	☐	☐
...det känns krävande?	☐	☐	☐	☐	☐
…du har svårt att hinna med dina egna besök inom hälso- och sjukvården?	☐	☐	☐	☐	☐

24. Behöver du själv för din egen hälsa stöd eller vård?
T.ex. från familj/släkt, det offentliga eller frivilligorganisationer.

☐ Ja, jag behöver stöd och får det
☐ Ja, jag behöver stöd, men får det inte
☐ Nej, jag behöver inte stöd

25. Hur bedömer du…
Markera ett svar för varje fråga.

	Mycket gott	Ganska gott	Någorlunda	Ganska dåligt	Mycket dåligt
…ditt allmänna hälsotillstånd?	☐	☐	☐	☐	☐
…ditt fysiska hälsotillstånd?	☐	☐	☐	☐	☐
…ditt psykiska hälsotillstånd?	☐	☐	☐	☐	☐

Tack för din medverkan!

KLIMAT NEUTRALT FÖRETAG
VERIFIERAT AV respect
WWW.KLIMATNEUTRAL.SE
Tryckt hos ett klimatneutralt företag Edita Bobergs AB

MILJÖMÄRKT
3041 0359
Trycksak

FSC
www.fsc.org
MIX
Papper från ansvarsfulla källor
FSC® C102746

SCB23011_01 April 2023

Informal care, assistance, or support for a family member/friend

Introduction and background to the survey

What is the purpose of this survey?

This survey aims to provide a picture of those who regularly provide care, assistance or support to a loved one due to physical or mental illness, disability or old age.

I do not provide care, assistance or support: should I answer the survey?

Yes, your answers are important even if you do not provide care or support to a family member, but it is only a few questions and takes about 3 minutes.

What do we mean by "family member/friend"?

By family member/friend, we mean someone in your family, relatives or someone else with whom you have a close relationship such as a friend, neighbor or colleague.

What do we mean by " informal care, assistance or support"?

By informal care, assistance or support we mean things you do on a fairly regular basis for someone with a disability, long-term illness or other needs. You may be caring for, helping or supporting your partner, a child, other family members, relatives or friends. Think about any type of care, help and support you provide to someone, beyond the usual care of children without special needs. This could be personal care or supervision. It could be more practical tasks such as shopping, giving someone a lift, housekeeping and home maintenance, or help in dealing with authorities or health professionals. It can also include help with finances, mail, bills, etc. or breaking social isolation. These are just some examples of providing care, help or support.

In the questions below, we use the words "care or support" as an umbrella term for all forms of care, help or support you provide.

What kind of help or support is not included?

This survey does not cover the help, care or support that you provide in your profession, for example as a care worker, personal assistant or guardian. The survey is only about the care, assistance and support you provide as a private individual. The survey does not cover the care that people give to children who do not have special needs.

NOTE! We ask that only you who have personally received this letter answer the survey and answer only for yourself.

By responding to this survey, you give consent for the information provided to be used for research purposes by researchers at the Aging Research Center, Karolinska Institutet and the National Competence Center for Caregivers. Your data will be depersonalized and treated confidentially, and your name will not appear in research publications or reports.

1. **What year were you born?**

Write the year with four digits, for example 1986 or 2001.

Year of birth: ☐☐☐☐

2. **Are you male, female or have another gender identity?**

☐ Male
☐ Female
☐ Other

3. **What is your current main occupation?**

Only one option can be given. Please indicate the option that best suits you.

☐ I am retired

☐ I work ☐☐☐ % of full time

☐ I am retired, but I work ☐☐☐ % of full time

☐ I work, but I have a sick leave of ☐☐☐ % of full time

☐ I do not work, I stay at home (not retired)

☐ Other, please write: ________

4. **Do you regularly (i.e. not just temporarily for a short period) provide care or support to one or more persons close to you?**

By care or support we mean everyday tasks, personal care or other support needed due to physical or mental illness, disability or age. Please read the introduction again if you are not sure if you belong to this group!

☐ Yes, one person
☐ Yes, two people
☐ Yes, three people
☐ Yes, more than three people

☐ No → *Go to question 25*

**5.**	**How often do you provide care or support to a related person (to one or more persons)?**<br>☐ Every day<br>☐ At least once <u>a week</u><br>☐ At least once <u>a month</u><br>☐ Less often than once a month ⟶ *Go to question 25*
**6.**	**Approximately how many hours on average per week do you provide care or support to a related person (to one or more persons in total)?**<br>☐ Less than 1 hour per week<br>☐ 1–10 hours per week<br>☐ 11–29 hours per week<br>☐ 30–59 hours per week<br>☐ 60 hours or more per week

Here are some questions about the person to whom you provide care or support. If you provide care or support to more than one person, please think about the person you provide the most care or support to.

**7.**	**Who is the person you care for or support <u>the most</u>?**<br>☐ Spouse/partner<br>☐ Child<br>☐ Parent<br>☐ Sibling<br>☐ Relative<br>☐ Friend, neighbour, acquaintance
**8.**	**Do you provide care or support to more than one person?**<br><br>You can select several options.<br>☐ No, I only provide support to a person indicated in the previous question.<br>☐ Yes, also spouse/partner<br>☐ Yes, also child<br>☐ Yes, also parent<br>☐ Yes, also sibling<br>☐ Yes, also relative<br>☐ Yes, also friend, neighbor, acquaintance
**9.**	**What is the gender of the person to whom you provide <u>the most</u> care or support?**<br>☐ Male<br>☐ Female<br>☐ Other
**10.**	**Does the person to whom you provide <u>the most</u> care or support speak Swedish?**<br>☐ Yes, fluently<br>☐ Yes, partly<br>☐ No, not at all

11. How old is the person to whom you provide the most care or support?

If you are not sure, make an estimate.

☐☐☐ Years

If you are unsure of age, you can also enter year of birth.

Year of birth : ☐☐☐☐

12. What type of accommodation does the person to whom you provide the most care or support have?

- ☐ Ordinary housing (villa or apartment)
- ☐ Sheltered housing, “seniorboende”
- ☐ Sheltered housing, “servicebostad”
- ☐ Special housing, group housing, nursing and care homes

13. Where do you live in relation to the person to whom you provide the most care or support?

- ☐ In the same household
- ☐ In different households but same building
- ☐ Within walking distance
- ☐ Not within walking distance, but within 30 minutes one way
- ☐ Between 30 minutes and 1 hour one way
- ☐ More than 1 hour one way

14. What is the main reason why the person to whom you provide the most care or support needs your help?

Only one option can be given. If the person has more than one health problem, you can indicate this in question 15.

- ☐ Alzheimer's disease, dementia, memory problems
- ☐ Cancer
- ☐ Stroke
- ☐ Heart attack, heart failure or other heart disease
- ☐ Diabetes
- ☐ Lung problems (COPD, asthma)
- ☐ Parkinson's disease or other neurological problems (e.g. epilepsy)
- ☐ Kidney disease (e.g., dialysis)
- ☐ Fracture (e.g. hip fracture after a fall)
- ☐ Physical disability
- ☐ Problems with vision or hearing
- ☐ Loneliness, social isolation
- ☐ Mental health problems (e.g. depression, schizophrenia)
- ☐ Abuse of alcohol or drugs
- ☐ Other reasons, explain in your own words:

15. Are there other reasons why the person you provide the most care or support to needs your help?

You can mark several options.

- ☐ No, there are no additional reasons
- ☐ Alzheimer's disease, dementia, memory problems
- ☐ Cancer
- ☐ Stroke
- ☐ Heart attack, heart failure or other heart disease
- ☐ Diabetes
- ☐ Lung problems (COPD, asthma)
- ☐ Parkinson's disease or other neurological problems (e.g. epilepsy)
- ☐ Kidney disease (e.g., dialysis)
- ☐ Fracture (e.g. hip fracture after a fall)
- ☐ Physical disability
- ☐ Problems with vision or hearing
- ☐ Loneliness, social isolation
- ☐ Mental health problems (e.g. depression, schizophrenia)
- ☐ Abuse of alcohol or drugs
- ☐ Other reasons, explain in your own words:

Here are some questions about the care or support you provide.

16. Thinking about a typical day when you provide care or support (to one or more people), approximately how much time do you spend on the following tasks?

	Hours	Minutes	Not relevant
Help with toileting, eating, dressing, personal care, walking and showering/bathing	☐☐	☐☐	☐
Grocery shopping, cooking, household chores, laundry, transportation, medication and managing finances	☐☐	☐☐	☐
Supervision (i.e. ensuring, reminding, motivating, supporting and preventing hazards)	☐☐	☐☐	☐
Socializing, providing social or emotional support	☐☐	☐☐	☐
Medical assistance (e.g. wound care, injections, help with rehabilitation)	☐☐	☐☐	☐
Coordinate care, contact health and/or social services (elderly care)	☐☐	☐☐	☐

17. Thinking about a <u>typical week</u> when you provide care or support (to one or more people), approximately how many days do you spend on the following tasks?

	Days per week	Not relevant
Help with toileting, eating, dressing, personal care, walking and showering/bathing	☐	☐
Grocery shopping, cooking, household chores, laundry, transportation, medication and managing finances	☐	☐
Supervision (i.e. ensuring, reminding, motivating, supporting and preventing hazards)	☐	☐
Socializing, providing social or emotional support	☐	☐
Medical assistance (e.g. wound care, injections, help with rehabilitation)	☐	☐
Coordinate care, contact health and/or social services (elderly care)	☐	☐

18. Is there anyone else besides you who cares for or supports the person you help <u>the most</u>?

You can mark several options.

☐ Yes, family members, relatives and/or friends

☐ Yes, elderly care such as home help, day care, special housing etc. (from the municipality or a private company)

☐ Yes, health care, home health care

☐ Yes, waiting service or other activities by voluntary organizations

☐ Yes, privately purchased help (e.g. cleaning service)

☐ No

19. a. How long have you been providing care or support to the person you currently help <u>the most</u>?

☐ 0-3 months

☐ 4-6 months

☐ 7 months-1 year

☐ 2-5 years

☐ 6-10 years

☐ More than 10 years

b. How long have you been providing care or support to someone else before (indicate the total time if you have provided care to more than one person)?

☐ I have not cared for anyone other than this person.

☐ 0-3 months

- ☐ 4-6 months
- ☐ 7 months-1 year
- ☐ 2-5 years
- ☐ 6-10 years
- ☐ More than 10 years

Here are some questions about different types of support you may need or receive. These questions relate to the total care you provide (to one or more people).

20. Have you received and/or been offered support from your municipality or other organizations in the form of...

Mark one answer for each question.

	Yes	Yes, was offered but refused	No, but would like to have it	No, and not interested
...information and advice?	☐	☐	☐	☐
...education/training?	☐	☐	☐	☐
...personal support call?	☐	☐	☐	☐
...support group?	☐	☐	☐	☐
...feel good activities (e.g. Nordic walking, aqua gym, massage)?	☐	☐	☐	☐
...financial contribution or financial support?	☐	☐	☐	☐
... health screening and health advice?	☐	☐	☐	☐
... respite care (at home or via day care, short-term or alternate care)?	☐	☐	☐	☐
... support via modern technology or the Internet (e.g. web information, video conference, online groups, GPS system)?	☐	☐	☐	☐
... support that facilitates work (possibility of teleworking, flexible working hours, communicating with managers via internet or similar)?	☐	☐	☐	☐

21. Do you personally feel that you, as a caregiver, receive enough support from...

	Yes, absolutely	Yes, mostly	No	I don't need/want it
…elderly care (äldreomsorgen)?	☐	☐	☐	☐
…healthcare?	☐	☐	☐	☐
… voluntary organizations?	☐	☐	☐	☐
… family/friends/neighbors?	☐	☐	☐	☐

Here are some questions about your situation as a carer, your feelings and your health. These questions relate to the overall care you provide (to one or more people).

22. In the last month, how much would you estimate that your sleep is disturbed because of the care or support you provide?

- ☐ My sleep is not disturbed at all
- ☐ About 1 night per week

- ☐ Between 2 and 3 nights per week
- ☐ Between 4 and 6 nights per week
- ☐ Every night

23. If you think about the care and support you provide overall, how often does it mean that...

	Always/almost always	Often	Sometimes	Rarely	Don't know/don't want to answer
... you find it difficult to spend time with your friends?	☐	☐	☐	☐	☐
... you experience loneliness?	☐	☐	☐	☐	☐
... you find it difficult to find time to exercise, such as taking a walk or going to the gym?	☐	☐	☐	☐	☐
... it is mentally stressful for you?	☐	☐	☐	☐	☐
... it is physically demanding for you?	☐	☐	☐	☐	☐
... you experience problems in your relationship with the person(s) you provide care or support to?	☐	☐	☐	☐	☐
... you have financial problems?	☐	☐	☐	☐	☐
... you experience a sense of satisfaction?	☐	☐	☐	☐	☐
... you experience problems in your relationships with family members?	☐	☐	☐	☐	☐
... it feels demanding?	☐	☐	☐	☐	☐
… you find it difficult to keep up with your own health care appointments?	☐	☐	☐	☐	☐

24. Do you need support or care for your own health?

E.g. from family/relatives, public or voluntary organizations.

☐ Yes, I need support and get it
☐ Yes, I need support but don't get it
☐ No, I do not need support

25. How do you assess...

Mark one answer for each question.

	Very good	Quite good	Okay	Quite bad	Very bad
… your general state of health?	☐	☐	☐	☐	☐
… your physical state of health?	☐	☐	☐	☐	☐
… your mental health?	☐	☐	☐	☐	☐

Thank you for your participation!
